# A Robust Dual-Stage Learning-Based Pipeline for Multiclass Segmentation of Multiple Sclerosis Lesions in MRI

**DOI:** 10.3390/diagnostics16152427

**Published:** 2026-07-31

**Authors:** Reza Naghne, Mahdiyeh Rahmani, Ali Kazemi, Mostafa Abdolghaffar, Tina Anjomshoa, Niusha Ghadesi, Abolfazl Zamanirad, Asra Karami, Ebrahim Najafzadeh, Parastoo Farnia

**Affiliations:** 1 Medical Physics and Biomedical Engineering Department, Faculty of Medicine, Tehran University of Medical Sciences, Tehran 1417653761, Iran; rnaghne@razi.tums.ac.ir (R.N.); mrahmani@razi.tums.ac.ir (M.R.); kazemi-a@razi.tums.ac.ir (A.K.); t_anjomshoa@razi.tums.ac.ir (T.A.); n-ghadesi@razi.tums.ac.ir (N.G.); a-zamanirad@razi.tums.ac.ir (A.Z.); 2 Research Center for Intelligent Technologies in Medicine (RCITM), Advanced Medical Technologies and Equipment Institute (AMTEI), Tehran University of Medical Sciences, Tehran 1417653761, Iran; 3 Research Center of Biomedical Technology and Robotics (RCBTR), Advanced Medical Technologies and Equipment Institute (AMTEI), Tehran University of Medical Sciences, Tehran 1417653761, Iran; 4 Research and Development Department, Parseh Intelligent Surgical Systems Company (Parsiss), Tehran 1418693913, Iran; a.mostafa.me@gmail.com; 5 Department of Molecular Imaging, Faculty of Advanced Technologies in Medicine, Iran University of Medical Sciences, Tehran 1449614535, Iran; karami.as@iums.ac.ir; 6 Finetech in Medicine Research Center, School of Medicine, Iran University of Medical Sciences, Tehran 1449614535, Iran; 7 Medical Physics Department, School of Medicine, Iran University of Medical Sciences, Tehran 1449614535, Iran

**Keywords:** multiple sclerosis, deep learning, machine learning, classification, segmentation, MRI, FLAIR, nnU-Net, UNETR++

## Abstract

**Background**: Accurate segmentation and classification of multiple sclerosis (MS) lesions are vital for a reliable diagnosis and disease monitoring. However, lesion heterogeneity in size, location, and intensity poses significant challenges to automated analysis. **Methods**: To address this, we developed a dual-stage pipeline integrating deep learning (DL) for precise spatial delineation and machine learning (ML) for robust classification of MS lesions. Two advanced DL models, nnU-Net and UNETR++, were optimized for lesion segmentation. Moreover, UNETR++ and several conventional ML methods were considered for the classification task, and Random Forest was found to be the best choice. **Results**: Experimental results indicate that nnU-Net outperformed UNETR++ for lesion segmentation across all cases, achieving a maximum improvement of 12.8%. During classification, Random Forest consistently outperformed advanced DL models, achieving at least 12% higher performance. Under practical conditions, an optimized hybrid pipeline that integrates nnU-Net for precise segmentation with Random Forests for robust classification delivers the best overall performance. Furthermore, qualitative analysis indicates that some apparent false positives may correspond to lesions missed during annotation, highlighting potential limitations in ground truth labeling. **Conclusions**: Overall, the proposed pipeline effectively leverages the complementary strengths of DL and ML, offering a promising, accurate framework for automated MS lesion analysis with potential clinical utility.

## 1. Introduction

Multiple sclerosis (MS) is a chronic neurodegenerative disease affecting approximately 2.5 million people worldwide [1]. The disease causes lesions in various regions of the brain, leading to a wide range of neurological symptoms. Visual and auditory disturbances, such as double vision or hearing loss, are common manifestations in patients with MS [2]. In addition to its physical consequences, MS also has social and mental impacts, making accurate and early diagnosis critically important [1]. Precise diagnosis of MS remains challenging, as it must be differentiated from other neurological disorders that mimic MS, such as acute disseminated encephalomyelitis and neuromyelitis optica spectrum disorder [3]. A key factor in MS diagnosis is the spatial distribution of lesions within the central nervous system. According to the 2017 revisions of the McDonald criteria, demonstrating dissemination in space (DIS) requires the observation of lesions in at least two of four specific anatomical areas: three brain regions (periventricular, juxtacortical/cortical, and infratentorial) and one spinal cord region [4,5]. Identifying lesion location can be of great importance, as it and severity are two factors that strongly influence clinical symptoms and disease progression [6]. Therefore, detection of lesions anywhere in the central nervous system is the first step toward MS diagnosis [7].

Magnetic resonance imaging (MRI) plays a critical role in demonstrating the dissemination of lesions across different brain regions and monitoring the development of new lesions over time [5]. Furthermore, the opportunity to observe new or acute hyperintense lesions alongside chronic lesions makes this modality a strong choice for follow-up and assessment of disease progression [5]. MS lesions are typically hyperintense on T2-weighted, proton density, and fluid-attenuated inversion recovery (FLAIR) sequences. These sequences have visual priority over the T1 sequence, which demonstrates the lesions as iso- or hypo-intense. However, pinpointing the exact number and location of lesions, considering their dispersion, remains a challenge.

In this manner, MS lesion segmentation is a necessary step before quantitative analysis of disease progression and estimation of destruction. Following segmentation, lesion classification by anatomical location is a critical step in treatment planning and disease monitoring [8,9]. Neuroradiologists commonly do MS lesion segmentation manually; however, this process is time-consuming, error-prone, and subject to inter-observer variability. Furthermore, determining the precise anatomical regions associated with each lesion can introduce additional uncertainty, potentially affecting clinical decision-making [10].

In recent years, artificial intelligence (AI) approaches have assisted many physicians in disease diagnosis. AI-based approaches for automatic MS lesion detection have significantly evolved from conventional image processing methods to machine learning (ML) algorithms and, more recently, deep learning (DL) models [8,11,12]. Despite extensive studies using various DL and ML approaches for MS lesion detection and segmentation, ongoing challenges remain in developing an automatic, highly accurate, and efficient diagnostic approach [13]. Motivated by these challenges, this study proposes an automated, robust pipeline for the segmentation and classification of MS lesions, with particular emphasis on lesion location, a critical factor in clinical decision-making. The proposed pipeline integrates DL–based and ML–based approaches, enabling accurate lesion identification and categorization. The first step involves using DL models to segment MS lesions from brain MRI images, which excel at capturing local, spatial, and pixel-wise features, thereby ensuring precise lesion detection.

Subsequently, the detected lesions are classified using both ML and DL algorithms that explicitly incorporate lesion location information. The proposed pipeline aims to facilitate faster, more accurate MS diagnosis, support treatment planning, and improve predictive assessment by addressing the challenges posed by the complex spatial characteristics of MS lesions across different brain regions.

## 2. Related Works

Artificial intelligence algorithms have been extensively applied to the segmentation and classification of MS lesions. In this section, previous studies on MS lesion segmentation and classification are discussed separately.

In 2015, Maier et al. [14] employed the random forest algorithm for MS lesion segmentation using voxel-by-voxel classification on multitemporal MRI images. Although their approach demonstrated improved performance on some metrics, the results appear constrained by an upper limit, and the models’ generalizability to unseen data was not adequately established. To address the limitations commonly associated with ML algorithms, Afzal et al. [15] proposed a Convolutional neural network (CNN) based method, achieving a Dice score of up to 0.67. However, their method had difficulty detecting lesions near the cortex or those close together.

CNN-based models offered notable improvements, and subsequent studies identified U-Net architectures as a more effective alternative for MS lesion segmentation [16,17]. To improve U-Net performance, several modifications to the standard U-Net architecture have been proposed. Alessia Rondinella et al. [18] incorporated a convolutional long short-term memory layer and an attention mechanism to enhance temporal and spatial feature representations. Similarly, Beytullah Sarica et al. [19] proposed a dense residual U-Net combined with an attention gate, efficient channel attention, and Atrous spatial pyramid pooling. Joshi et al. [20] combined a CNN with a graph convolutional network, which poses a high computational burden.

Additionally, Ton-Wiltgen et al. [21] proposed a pipeline comprising three 3D U-Net models trained on 3T FLAIR and T1 training images, employing a combination of binary cross-entropy and Tversky loss functions for white matter lesion segmentation. Placidi et al. [22] demonstrated that dual U-Net models applied to 2D FLAIR slices, incorporating uncertainty as an intermediate class, can produce reliable 3D lesion masks without the need for multiple contrasts. In parallel, Ghosal et al. [23] employed two distinct U-Net architectures: one emphasizing residual concatenation and the other spatial channel attention to segment lesions as localized objects. Despite promising results, their approach had limitations in capturing spatial relationships when lesions spanned multiple slices.

Overall, recent studies indicate that U-Net variants achieve robust and reliable performance in MS lesion segmentation. However, developing a precise, lightweight network capable of maintaining high performance across varying conditions, such as low magnetic field strength and limited MRI contrast, remains a clinically significant and open research challenge.

In addition to the importance of accurate lesion segmentation, determining the anatomical location of lesions is essential. In this context, numerous studies have investigated the problem of MS lesion classification. Ying Wu et al. [24] conducted a study for automatically segmenting MS lesions into three subtypes (i.e., enhanced lesions, T1 black holes, and T2 high-intensity lesions) using a region-growing algorithm combined with a k-NN classifier. However, these traditional methods rely largely on clinical judgment and the interpretation of MRI characteristics, such as lesion location and signal intensity. These approaches often suffer from limitations, including reduced speed, lower accuracy, limited repeatability, and difficulty distinguishing active from inactive lesions [25]. Such challenges have motivated the adoption of ML-based solutions. Decision trees (DTs), random forests (RFs), boosting, and support vector machines (SVMs) are among the most commonly used ML algorithms for MS lesion classification [26,27,28,29]. For instance, Christos P. Loizou and colleagues [30] applied SVM models to classify normal white matter changes in MS patients. At the same time, Farshad Shekari et al. [26] used SVM to differentiate active from inactive lesions. More recently, DL techniques have gained considerable attention for MS lesion detection and classification.

Francesco La Rosa et al. [31] employed a 3D U-Net to detect and classify cortical MS lesions into leukocortical, subpial, and intracortical categories using 7 Tesla MRI scans. However, reliance on ultra–high–field MRI limits the model’s generalizability to more commonly used scanners, such as 1.5T or 3T systems. Similarly, Rostami et al. [32] proposed a radiomics-based sequential DL pipeline to classify active versus non-active lesions using T2-weighted images, demonstrating that a single contrast image can be beneficial and reduce the need for contrast agent injection. Furthermore, Davanian et al. [33] reported the outcomes of the top two teams in the MS lesion segmentation and localization challenge across different intensity levels, which correspond to different brain regions. Both teams implemented a U-Net-based architecture, with one incorporating Long Short-Term Memory (LSTM) and the other using variable-sized convolutional filters to improve segmentation performance. Overall, U-Net architectures have demonstrated superior performance in medical image segmentation by capturing both local and contextual features. Nevertheless, significant challenges remain in accurately detecting, segmenting, and, specifically, classifying MS lesions. Despite its clinical importance, lesion classification has received comparatively less research attention than segmentation tasks. Therefore, further studies are required to develop more robust and clinically applicable approaches. In this study, we propose a pipeline that integrates optimized U-Net models with learning-based classifiers and post-processing techniques to enable flexible and automated segmentation and classification of MS lesions. The proposed approach aims to improve diagnostic support and facilitate more accurate clinical assessment of MS lesions.

## 3. Materials and Methods

**Figure 1:**
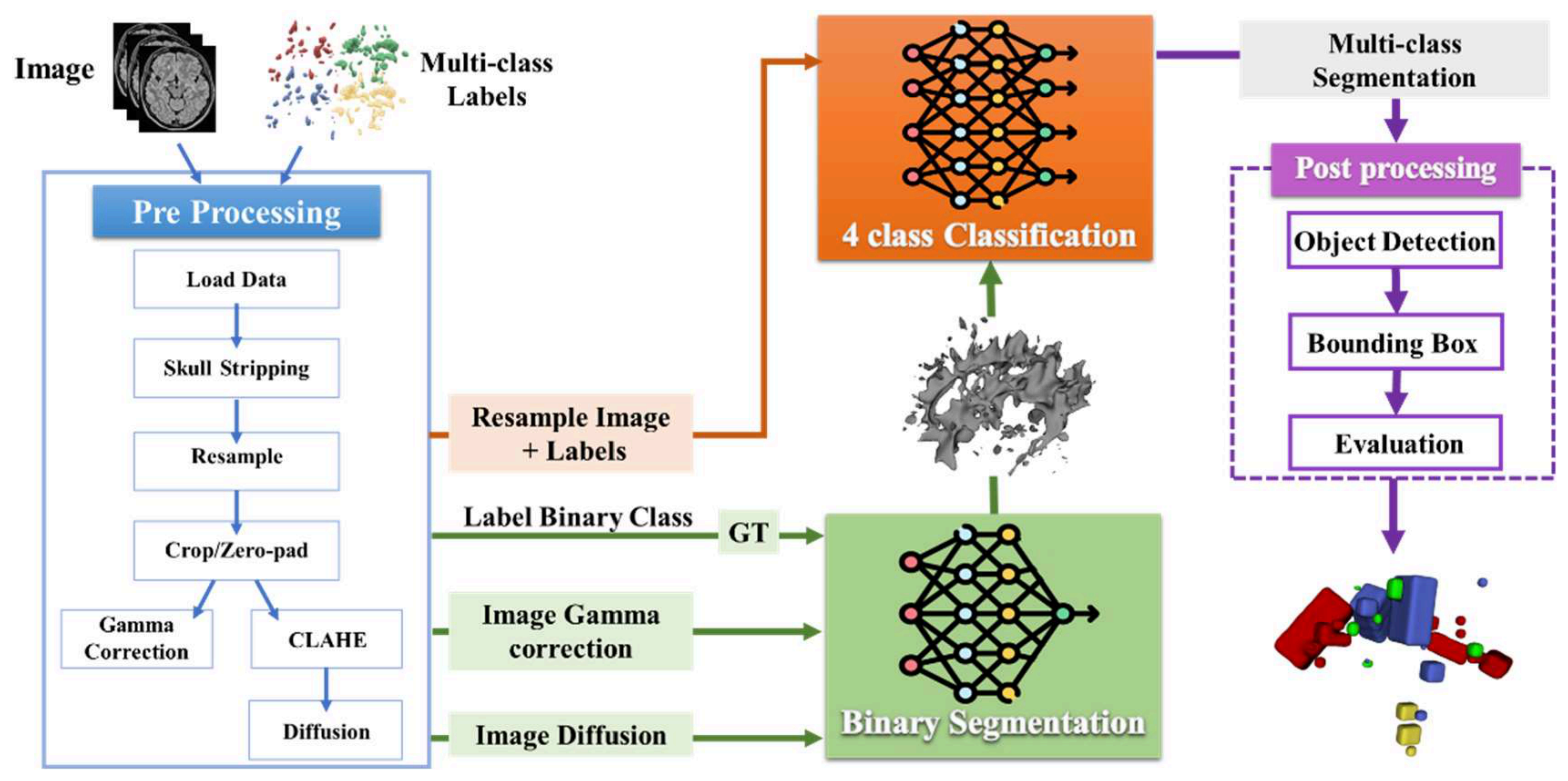
Block diagram of the execution stages, which are performed in four steps: preprocessing, binary segmentation of lesions, four-class lesion classification, and post-processing.

We propose an automated MS lesion-detection pipeline that segments and classifies lesions based on their intrinsic and spatial properties. In the following, as shown in Figure 1, we describe data preparation, lesion segmentation and classification, and post-processing.

### 3.1. Dataset and Preprocessing

In this study, we used an MS dataset obtained from the IAI2023 challenge [33]. The dataset comprises FLAIR MRI scans from 81 patients, acquired on a 1.5-Tesla Siemens Avanto system. Each scan was provided as a 3D NIFTI volume with spatial dimensions of 160 × 256 × 236 voxels, offering high-resolution structural information. Alongside each MRI volume, a corresponding 3D mask of identical dimensions was provided to delineate lesion regions. The lesion ground truths are categorized into four distinct classes: periventricular, white matter, juxtacortical, and infratentorial zones. This classification enables detailed analysis of lesion distribution patterns across anatomical regions, which is essential for both clinical assessment and the development of automated lesion segmentation methods. Although a cohort of 81 patients is consistent with the sample sizes typically utilized in established neuroimaging benchmarks, which frequently range from 20 to 100 cases to validate advanced architectures [34,35,36,37,38,39,40,41], the dataset used in this study is uniquely valuable. To our knowledge, it is the only publicly available resource providing manually verified ground truth masks meticulously categorized into four distinct anatomical zones.

**Figure 2:**
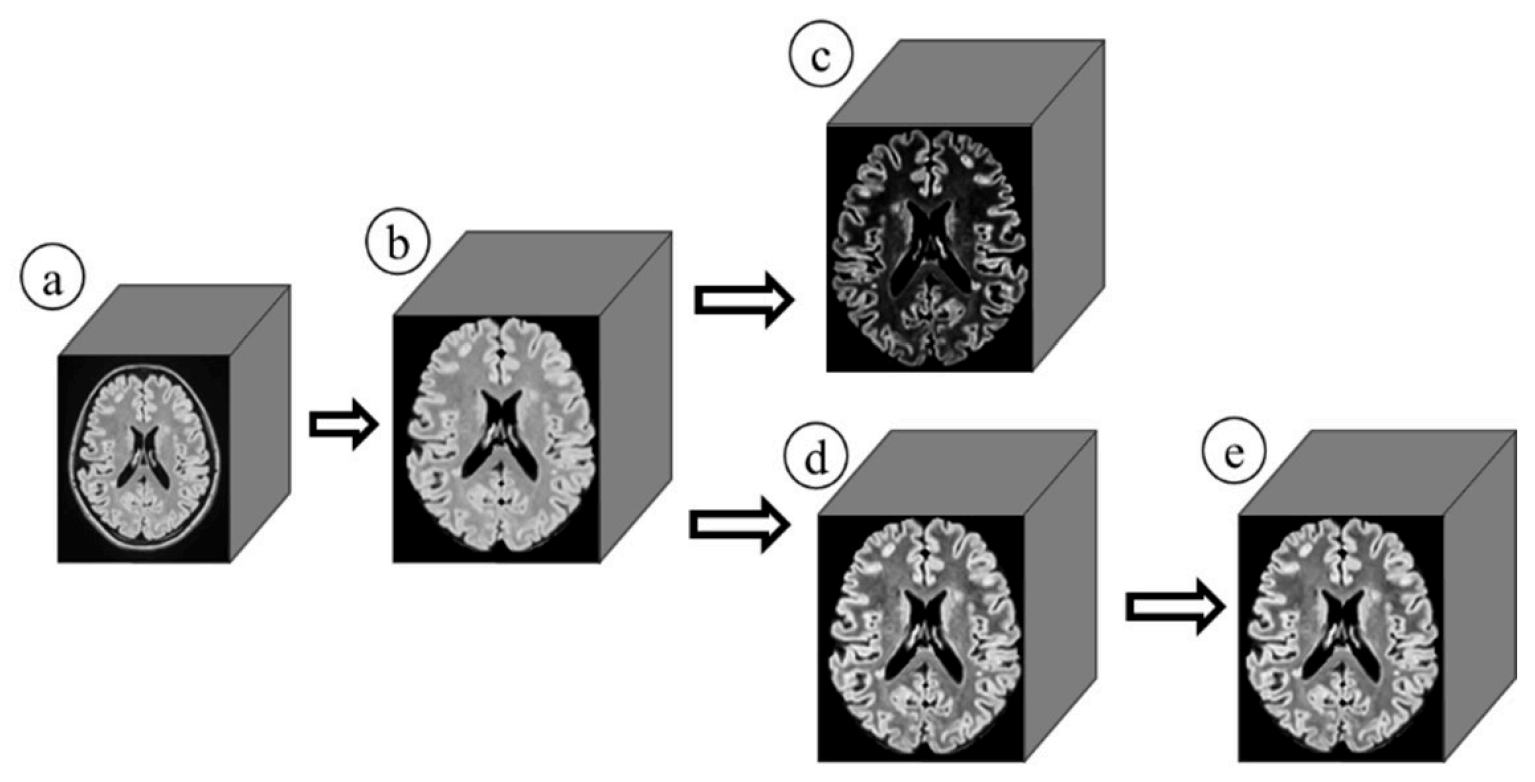
Illustration of the preprocessing steps, which are carried out as: (**a**) Input image, (**b**) Output of brain extraction, resampling, and cropping, (**c**) Output of gamma correction, (**d**) Output of CLAHE, and (**e**) Output of non-linear diffusion filtering.

The preprocessing pipeline, as shown in Figure 2, began with Brain Extraction. This ensured that the model concentrated exclusively on clinically relevant anatomy. Afterward, the images were resampled to an isotropic voxel spacing of 0.8 × 0.8 × 0.8 mm and subsequently padded to a fixed size of 256 × 256 × 256. Based on the lesion intensity characteristics (summarized in Table 1), two enhancement techniques were applied in parallel to each volume. To establish a highly rigorous evaluation framework, all hyperparameters, including those for Gamma correction and CLAHE, were tuned exclusively on the training set to prevent data leakage. A comprehensive description of the specific parameter settings applied during these preprocessing steps is provided in Section 3.7 (Experimental Settings).

### 3.2. MS Lesion Segmentation

**Figure 3:**
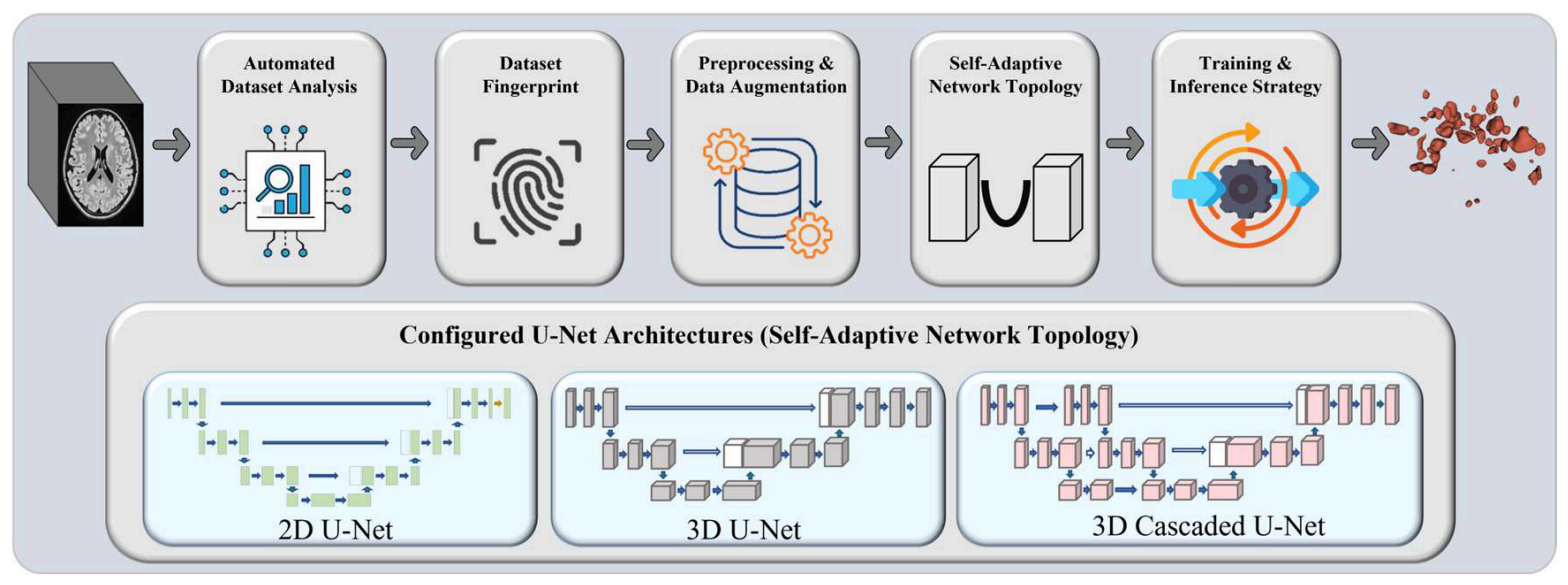
Schematic overview of the nnU-Net automated segmentation framework. The upper pipeline illustrates the automated configuration process, progressing from dataset analysis and fingerprint extraction to preprocessing, adaptive network topology configuration, and training strategies. The lower panel displays the three configured structures generated by the framework: 2D U-Net, 3D U-Net, and 3D Cascaded U-Net.

The first step in MS lesion detection after preprocessing is lesion segmentation. For this purpose, we optimized and composed two DL approaches: transformer- and CNN-based networks. To ensure a fair comparison, we selected two networks with balanced capabilities: nnU-Net and UNETR++, which are described in detail below. We applied nnU-Net [13], developed by Isensee et al. in 2021 (Figure 3). The nnU-Net is a DL pipeline that automates segmentation and includes critical topological parameters of the network architecture. A key feature of nnU-Net is its ability to analyze dataset-specific characteristics and adjust its configuration accordingly. It first generates a dataset fingerprint that captures essential properties, such as image size, resolution, voxel spacing, and intensity distribution. Using this information, nnU-Net automatically determines an optimized pipeline by adapting the network architecture, patch size, and batch size while considering computational constraints. This systematic approach eliminates the need for manual adjustments, enabling the pipeline to adapt to diverse medical imaging tasks without requiring custom modifications. The nnU-Net supports multiple U-Net variants, including 2D, 3D, and cascaded 3D architectures. In this study, we employed the 3D pipeline to improve performance, as MS lesions are typically small and require precise volumetric analysis.

This self-configuring nature enables nnU-Net to achieve top performance across multiple segmentation challenges, including CT and MRI scans, without requiring extensive domain-specific training. It achieves high segmentation accuracy even with limited training data, making it an efficient and scalable solution for biomedical image analysis. Due to these characteristics, we have selected nnU-Net for our study.

**Figure 4:**
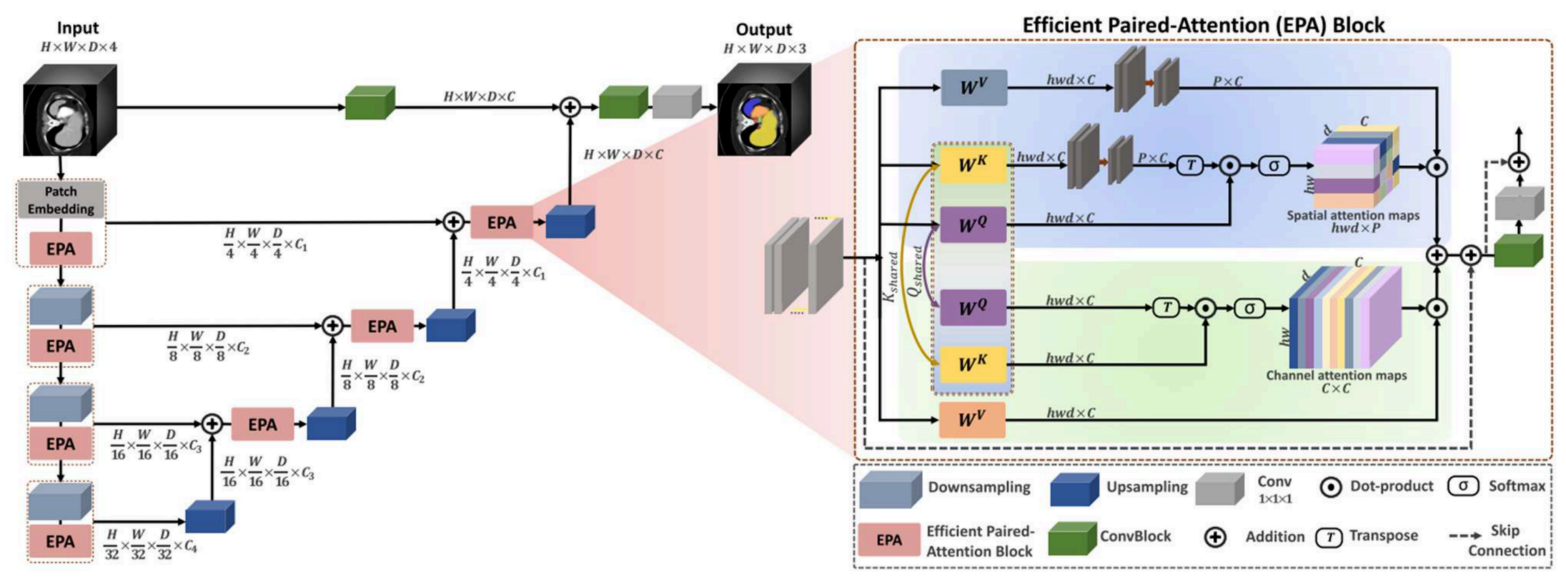
The illustration of the UNETR++ model with an encoder–decoder structure. As depicted in the EPA block diagram (**right**), the first (**upper**) attention module captures spatial dependencies, while the second (**lower**) attention module focuses on channel-wise dependencies. The outputs from both attention modules are subsequently fused and processed through convolutional blocks (Reprinted from [42] under a Creative Commons Attribution (CC BY 4.0) license).

Another network explored for MS lesion segmentation is UNETR++, which is designed to enhance the efficiency and accuracy of 3D medical image segmentation [42]. Unlike UNETR, which relies solely on high-complexity quadratic transformers, UNETR++ achieves linear complexity for spatial attention, balancing accuracy, efficiency, and computational cost. Additionally, it reduces floating-point operations per second (FLOPs) and the number of parameters, while achieving higher segmentation accuracy across multiple datasets. These advantages motivated our choice of UNETR++ for this task. UNETR++ introduces the Efficient Paired Attention (EPA) block, which effectively captures both spatial and channel-wise features through two interdependent (parallel) branches. These blocks operate via shared-key query mechanisms between two attention modules, enabling the learning of complementary extracted features and improving their representation. The network follows a 3D encoder–decoder architecture (Figure 4). The encoder begins with a patch embedding, in which the input volume is divided into non-overlapping patches of sizes *P*_1_ = 4, *P*_2_ = 4, and *P*_3_ = 2. The transformed input is then mapped using a kernel of size *C*, producing feature maps. After patch embedding, the encoder has three stages, each beginning with a convolution that reduces the feature map resolution by a factor of 2 before passing through an EPA block. The decoder contains four stages, with the first stage using transposed convolution for up-sampling, doubling the resolution. The subsequent stages contain an EPA block and up-sampling. Skip connections connect corresponding encoder and decoder stages to restore spatial information and improve accuracy. Finally, the output passes through two convolution layers (3 × 3 × 3 and 1 × 1 × 1) to generate the final predicted mask.

### 3.3. MS Lesion Classification

After MS lesion segmentation, it is important to categorize the lesions into four distinct classes. Leveraging feature engineering around lesions, we pursued two paths: a fully DL approach and a hybrid algorithm. Therefore, we would be able to perform pixel-wise classification using ML to maintain accuracy with limited data, and use DL techniques to achieve a fully automated pipeline. The machine learning-based approach operates at the object level (per-lesion), analyzing geometric and spatial feature vectors of isolated lesions via connected component analysis. Conversely, the deep learning-based approach (UNETR++) operates at the voxel level (per-pixel), assigning each lesion pixel to an anatomical class by leveraging local spatial relationships.

In this way, a 4-class classification experiment was carried out under three different input configurations, where the original data were paired with: (i) the ground truth lesion masks, (ii) the lesion masks predicted from the nnU-Net segmentation network, and (iii) the lesion masks predicted by the UNETR++ network. These three settings allowed us to assess the impact of segmentation quality on downstream lesion-type classification performance and to compare the behavior of the ML- and DL-based classifiers under identical conditions.

Classification based on ML algorithms relies on identifying attributes that differentiate between classes. In this study, classification is based on the anatomical features of MS lesions in brain MRI scans, where extracted features are categorized into two categories: (1) intensity- and geometry-based features (mean intensity, skewness, kurtosis, perimeter, and eccentricity) and (2) spatial location-based features (e.g., lesion centroid, bounding box, and center distance). These features capture the distribution of lesion intensity, spatial position, and geometric characteristics. These extracted features capture information about the spatial positions of lesions, their intensity levels, and their geometric shapes and sizes.

For the feature selection step, several algorithms, including Minimum Redundancy Maximum Relevance, Chi-square, Analysis of Variance (ANOVA), and T-test, were applied to identify the most relevant features and reduce dimensionality [43]. The final selected features included the lesion centroid, perilesional intensity statistics (25th, 50th, and 75th percentiles), spatial distribution metrics (minimum and maximum Euclidean distances from the image center), and lesion volume (voxel count). Feature selection and classifier optimization were performed solely on the training data, with the test set kept strictly separate to serve as an independent performance benchmark. Furthermore, these features were utilized for training several classifiers, including Random Forest for its ensemble nature and ability to reduce overfitting through bagging [44], Multilayer Perceptron for its capacity to handle non-linear relationships via hidden layers [45], K-Nearest Neighbors for its non-parametric approach based on similarity/proximity [46], Logistic Regression as a probabilistic and linear model [47], and Decision Tree for its hierarchical structure and interpretability [48]. In addition to ML-based classifiers, the DL architecture UNETR++ was used to classify lesions into four main categories. In this model, each pixel is classified based on local features and spatial relationships, enabling accurate identification of MS lesions across different brain regions.

### 3.4. Post-Processing

**Figure 5:**
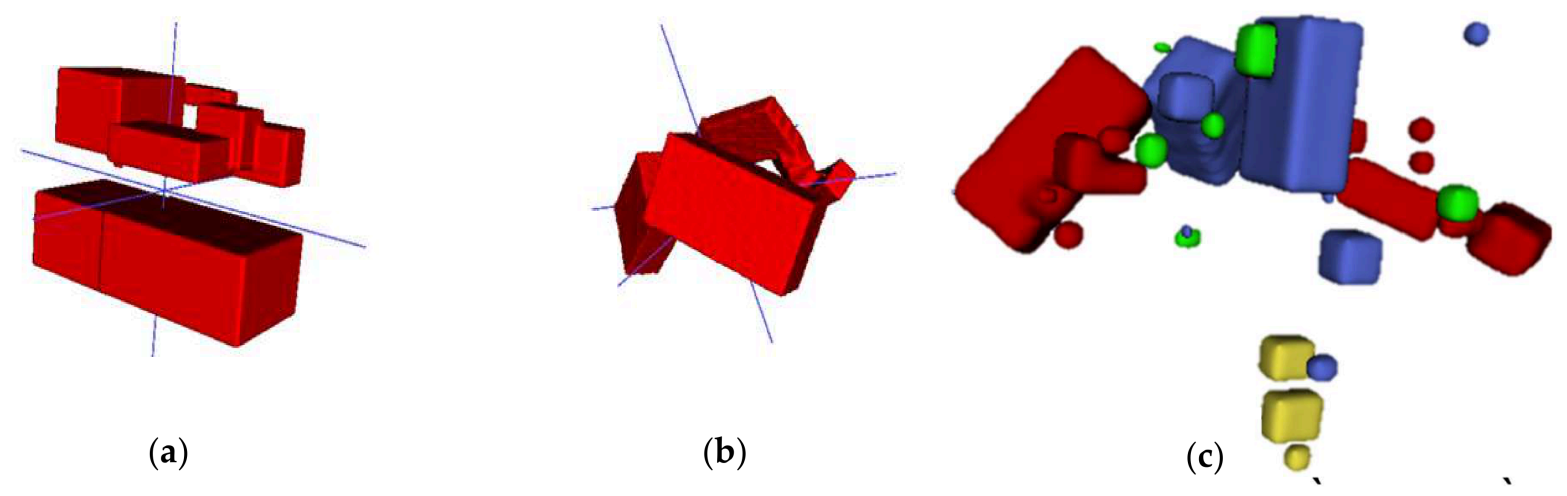
Illustration of bounding boxes. (**a**) simple bounding boxes (first method), (**b**) PCA-based bounding boxes (second method), (**c**) the bounding boxes of the combination method for each of the 4 classes: periventricular (red), white matter (green), juxtacortical (blue), and infratentorial (yellow).

The outputs of the preceding networks are processed to identify connected components within each lesion, corresponding to MS lesions. This step allows the extraction of intrinsic and spatial properties of each lesion relative to neighboring lesions. In this stage, bounding boxes are generated to determine the regions of interest for MS lesions. Therefore, the geometric structure of each lesion is analyzed by assessing point connectivity. Two approaches are employed to apply bounding boxes. In the first approach, conventional bounding boxes are placed in the image space based on the lesion’s coordinates and size, including its geometric center and dimensions. In the second approach, bounding boxes are aligned along the direction of the lesion’s principal eigenvector using Principal Component Analysis (PCA). The PCA-based method aligns bounding boxes with the lesion’s principal axes, resulting in a more precise fit. This process begins by computing the geometric center and covariance matrix to analyze the distribution of lesions [49]. The covariance matrix is then used to extract the eigenvalues and eigenvectors, which determine the primary directions of dispersion, as expressed in Equation (1) [50].

(1) $$cov*v_{i}=\lambda_{i}*\;v_{i}\;$$

where $\lambda_{i}$ is the eigenvalue, $v_{i}$ is the eigenvector, and cov is the covariance matrix. The lesion points are transformed into this new coordinate system to align the bounding box better. The minimum and maximum coordinates define the dimensions, ensuring a precise fit. Finally, the bounding box is mapped back to the original space, preserving its correct position and orientation. Figure 5 illustrates the bounding boxes generated by both methods.

### 3.5. Loss Function

To effectively train the segmentation network, a hybrid loss function was employed by combining Dice Loss and Cross-Entropy (CE) Loss (Equation (2)). This integrated approach ensures accurate region-level delineation while maintaining reliable pixel-wise classification performance, especially in the presence of class imbalance and hard-to-segment lesions. Dice Loss emphasizes the spatial overlap between the predicted and ground truth lesion areas, making it particularly suitable for imbalanced datasets, such as those encountered in MS lesion segmentation. The CE Loss (Equation (3)) complements this by providing a strong pixel-level classification signal, enabling accurate discrimination between lesion and non-lesion regions. In Equation (3), $t_{i}$ represents the target distribution, and the ${f(s)}_{i}$ denotes the predicted probability for i class. The predicted probability is obtained using the softmax function applied to the logits $s_{i}$. The total loss is defined as a weighted sum of the two loss components:

(2) $$L_{total}=\lambda_{Dice}\cdot L_{Dice}+\lambda_{CE}\cdot L_{CE}$$

(3) $$CE=-\sum_{i}^{c}t_{i}\log{\left(f(s\right)}_{i}),\;\;\;\;\;\;{f\left(s\right)}_{i}=\frac{e^{s_{i}}}{\sum_{j}^{c}e^{s_{j}}}\;\;$$

where $\lambda_{Dice}$ and $\lambda_{CE}$ are empirically determined weighting coefficients that balance the contributions of the Dice and CE losses. For binary segmentation, the weights are set to $\lambda_{Dice}=1.5$ and $\lambda_{CE}=0.2$, whereas for multiclass segmentation, they are set to $\lambda_{Dice}=0.6$ and $\lambda_{CE}=0.9$. This hybrid loss function enables the model to learn robust segmentation representations by jointly optimizing region- and voxel-based objectives, thereby improving detection of subtle and complex MS lesions.

### 3.6. Evaluation Metrics

Different evaluation criteria were used depending on the nature of the data. Missing a lesion is a major clinical error, as it can falsely indicate that the treatment is effective or the disease is stable when it is not. Also, in cases where removal of the lesion is necessary, even at the cost of destroying healthy tissue, using a sensitivity metric seems essential. This criterion is especially important for detecting small or deep structures. Higher sensitivity values indicate that the model effectively captures most of the target structures.

Different evaluation metrics were employed depending on the characteristics of the segmentation task. Missing a lesion represents a critical clinical error, as it may incorrectly suggest that the treatment is effective or that the disease is stable when it is not. In clinical practice, particularly when lesion removal is required even at the cost of affecting surrounding healthy tissue, high sensitivity is essential (Equation (4)). This metric is especially important for detecting small or deep lesions. Higher sensitivity values indicate that the model successfully identifies most true lesions.

(4) $$Sensitivity=\frac{TP}{TP+FN}$$

However, a model optimized solely for high sensitivity may produce many false positives. Therefore, the precision metric was also considered to reduce the number of incorrectly detected lesions, which may correspond to noise or normal brain structures. Improving precision reduces radiologists’ workload by limiting unnecessary false-positive detections (Equation (5)).

(5) $$\mathrm{Precision}\;=\frac{\mathrm{TP}}{\mathrm{TP}\;+\mathrm{FP}}$$

To simultaneously account for both sensitivity and precision, the Dice score coefficient (Equation (6)), which represents the harmonic mean of these two measures, was used. This score provides a more balanced measure of a model’s ability to detect lesions and the fidelity of its detections. It quantifies the volumetric overlap between the predicted and ground truth distributions.

(6) $$\mathrm{Dice}\;\mathrm{score}\;\mathrm{coefficient}=\frac{2\mathrm{TP}}{2\mathrm{TP}+\mathrm{FP}+\mathrm{FN}}$$

The Dice score tends to measure performance closer to the mean, with minor errors having little effect. To account for errors, the Intersection over Union (IoU) score (Equation (7)) was used, which approximates worst-case performance.

(7) $$IoU=\frac{TP}{TP+FP+FN}$$

It is worth noting that statistical metrics, such as specificity, which include true negative values in their calculations, were not evaluated due to the large background region.

Alongside voxel-wise metrics, lesion-wise evaluation is clinically essential. Since high sensitivity is often prioritized in MS lesion detection to avoid missing critical lesions, we explicitly report the Lesion-wise F1-score (equivalent to the Lesion-wise Dice score). This metric applies the harmonic mean formulation of Equation (6) using discrete lesion counts (TP, FP, FN) rather than voxel volumes. However, the evaluation continues with lesion-wise metrics, which can provide additional insight into the segmentation model’s performance. The following evaluation metrics focus on the distance between two volumes.

The 95th-percentile Hausdorff distance (HD95) quantifies the maximum boundary discrepancy while reducing sensitivity to outliers. HD95 computes the largest minimum distance between corresponding points on the predicted and ground truth surfaces (Equation (8)). In practice, this metric reflects the worst boundary mismatch for 95% of ground truth surface points.

(8) $$H_{95}\left(x,y\right)=\max\left(h_{95}\left(x,y\right),h_{95}\left(y,x\right)\right)$$

The $h_{95}$ represents the 95th-percentile value of the shortest two-way Euclidean distances computed from each point in one set to the closest point in the opposing set.

Because HD95 captures the worst-case error, small boundary variations can greatly affect its value, prompting reliance on more robust metrics such as ASD.

The Average surface distance (ASD) measures the mean boundary error between the ground truth and predicted surfaces, providing an estimate of overall boundary alignment (Equation (9)). This metric is also robust to outliers.

(9) $$ASD\left(A,\;B\right)=\frac{1}{\left|S_{A}\right|+\left|S_{B}\right|}\left(\sum_{a\in S_{A}}\underset{b\in S_{B}}{\min}d\left(a,b\right)+\sum_{b\in S_{B}}\underset{b\in S_{A}}{\min}d(b,a)\right)$$

where *A* denotes the ground truth and *B* the segmentation. $S_{A}$ and $S_{B}$ denote the sets of surface voxels corresponding to *A* and *B*, respectively, and d denotes the Euclidean distance. Except for what has been stated, a great concern is how the model will perform when exposed to new, unseen data. Therefore, to account for sample size constraints and provide a statistically robust evaluation of patient-level variance, bootstrapping was employed. Bootstrapping is a resampling technique that creates multiple pseudo datasets by sampling with replacement from the original dataset and then repeatedly evaluates the model on these resampled sets. The variation across these repeated evaluations provides an estimate of the model’s variance, stability, and uncertainty. Additionally, 95% confidence intervals (CIs) were computed. CIs show the range of values you anticipate the true estimate to fall between if the study is repeated numerous times.

### 3.7. Experimental Setup

To ensure strict reproducibility, all experiments were run on a Windows workstation equipped with an Intel Core i9-14900K, 64 GB of DDR5 RAM, and dual NVIDIA RTX 4090 GPUs (24 GB of VRAM each). The volumetric preprocessing was implemented via SimpleITK. Intracranial regions were isolated using an automated Brain Extraction Tool (BET). Raw volumes were isotropically resampled to 0.8 × 0.8 × 0.8 mm spacing and cropped/padded to a 256 × 256 × 256 matrix. Image intensities were adjusted using CLAHE (clip limit 2, 8 × 8 grid), followed by Gamma Correction (coefficient 3) and anisotropic diffusion filtering (gradient method, 10 iterations, conductance 0.3).

The nnU-Net architecture automatically configured a 6-stage 3D ResidualEncoderUNet (feature maps: [32, 64, 128, 256, 320, 320]). Training utilized Z-Score normalization, a patch size of 128 × 128 × 128, and a batch size of 2. Optimization was performed via SGD (learning rate 0.01, Nesterov momentum 0.99, weight decay 3 × 10^−5^) regulated by a PolyLRScheduler over 300 epochs.

For both the binary segmentation and the fully deep-learning-based classification tracks, the UNETR++ architecture shared identical core hyperparameters. The network used a 64 × 64 × 64 patch size, a batch size of 1, 4 attention heads, and channel dimensions of [32, 64, 128, 256]. It was trained for 300 epochs using the Adam optimizer (learning rate 0.0005, weight decay 1 × 10^−4^) with a hybrid loss function (Dice weighted at 1.5, Cross-Entropy at 0.2).

Dynamic 3D data augmentation applied during training included random rotations (±30°), isotropic scaling (0.7–1.4), gamma corrections (0.7–1.5), and Gaussian noise (variance 0–0.1). Explicit early stopping was omitted; models were trained for the full 300 epochs to fully explore the optimization landscape, with the best model selected based on validation performance. To generate binary masks, a fixed threshold of 0.5 was applied to the Softmax (nnU-Net) and Sigmoid (UNETR++) outputs.

For the random forest classification track, nine features were extracted per 3D connected component: three spatial centroids, three local intensity percentiles (25th, 50th, 75th; via a 7 × 7 × 7 dilated neighborhood), two Euclidean distances to the image center, and lesion volume. Features were standardized using a RobustScaler fitted exclusively on the training partition. An exhaustive Bayesian search spanning 1000 trials was conducted using k-fold cross-validation, with the optimal number of folds dynamically evaluated between 3 and 10. The search space rigorously evaluated the number of estimators (50 to 300), maximum tree depth (5 to 50), minimum samples required to split an internal node (2 to 20), minimum samples required to be at a leaf node (1 to 20), and the quality criterion (Gini impurity versus Shannon entropy).

Following model classification, an automated post-processing pipeline was implemented to isolate, optimize, and resolve overlaps among the predicted regions of interest (ROIs). Predicted multi-class masks were separated into spatial clusters using 3D connected-component labeling (26-connectivity). Standard and PCA-oriented bounding boxes were evaluated to maximize the component-level Dice score. Spatial collisions between neighboring same-class components were resolved via an automated morphological subtraction loop, where the smaller component was dilated (3 × 3 × 3 kernel) and subtracted from the larger one.

## 4. Results

To ensure rigorous evaluation, the dataset was partitioned strictly at the patient level, with 61, 10, and 10 cases allocated for training, validation, and testing, respectively. The test set (Table 2) was selected to ensure diversity in lesion counts across all four classes, ranging from cases with few lesions to those with numerous lesions. This approach was taken to minimize class imbalance bias and provide a comprehensive overview of the dataset.

**Figure 6:**
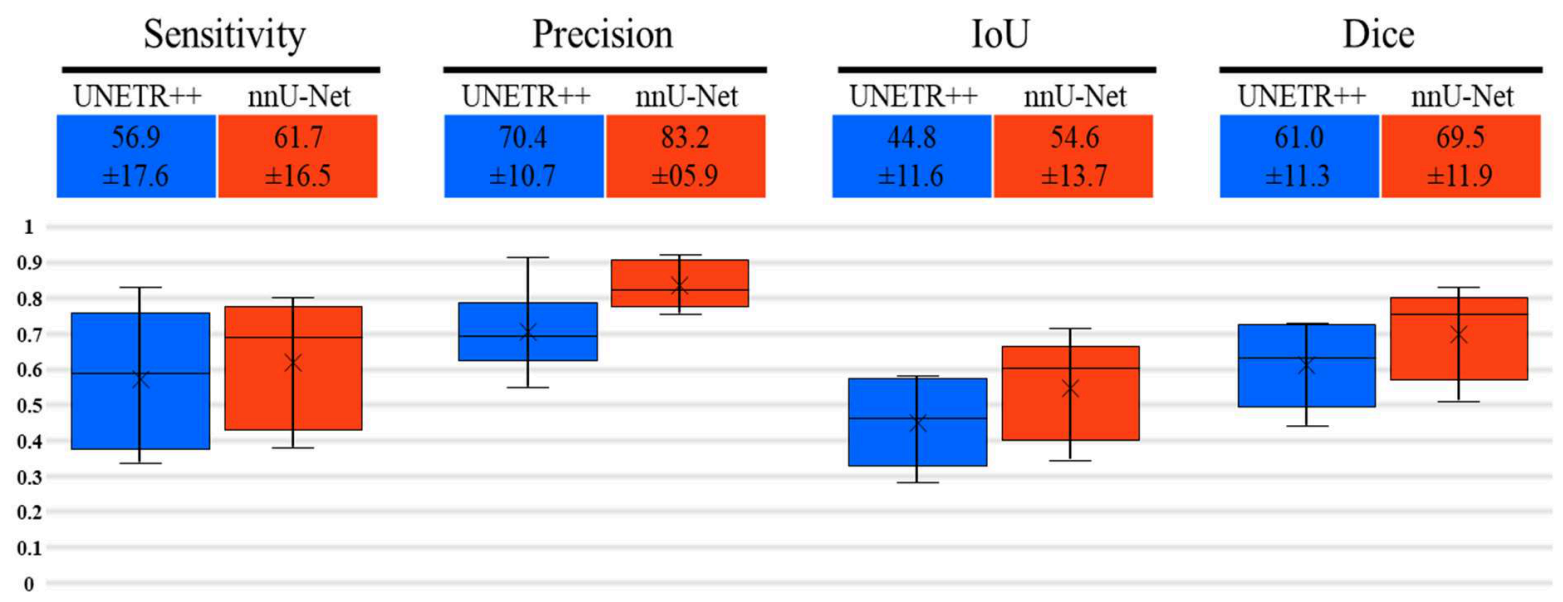
The calculated mean and std of four evaluation metrics (Sensitivity, Precision, IoU, and Dice) for binary segmentation outputs from UNETR++ and nnU-Net.

In this study, the segmentation performance of UNETR++ and nnU-Net was first investigated and compared, followed by the evaluation of the classification results. The binary segmentation results, plotted by pixel count, are shown in Figure 6. Overall, the nnU-Net network achieves a higher average score across all evaluated metrics. However, UNETR++ exhibited lower standard deviation (std) in IoU and Dice scores, indicating more stable performance across test cases.

**Figure 7:**
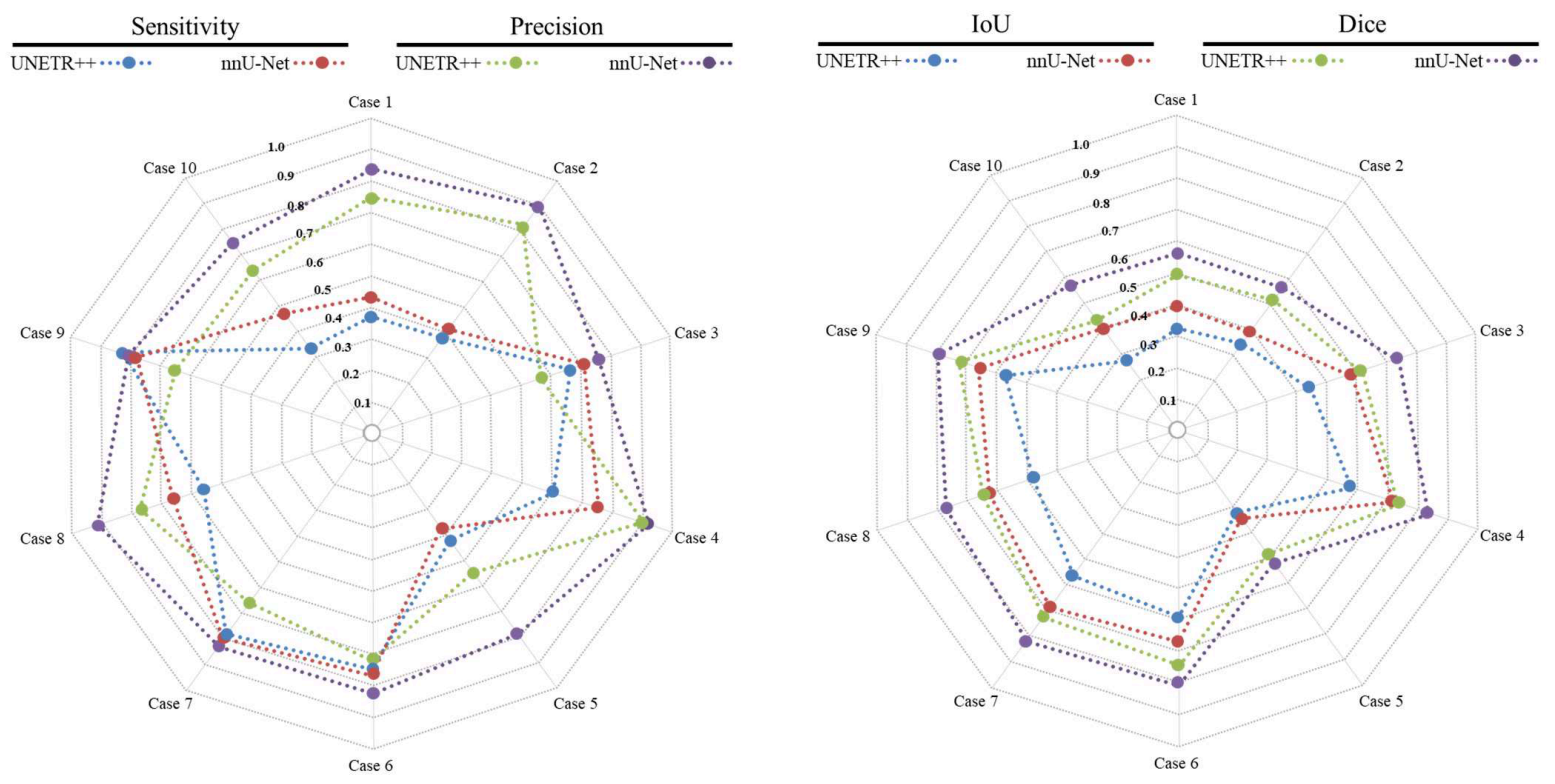
Results of four evaluation metrics (sensitivity, precision, IoU, and Dice) for the binary segmentation results of UNETR++ and nnU-Net across all test cases.

Also, since the test data is representative of the entire dataset, the results for each case are shown in Figure 7 to demonstrate consistency and identify which algorithm performs better across individual samples. The nnU-Net network achieves superior IoU and Dice scores across all test cases; however, sensitivity and precision vary by case.

**Figure 8:**
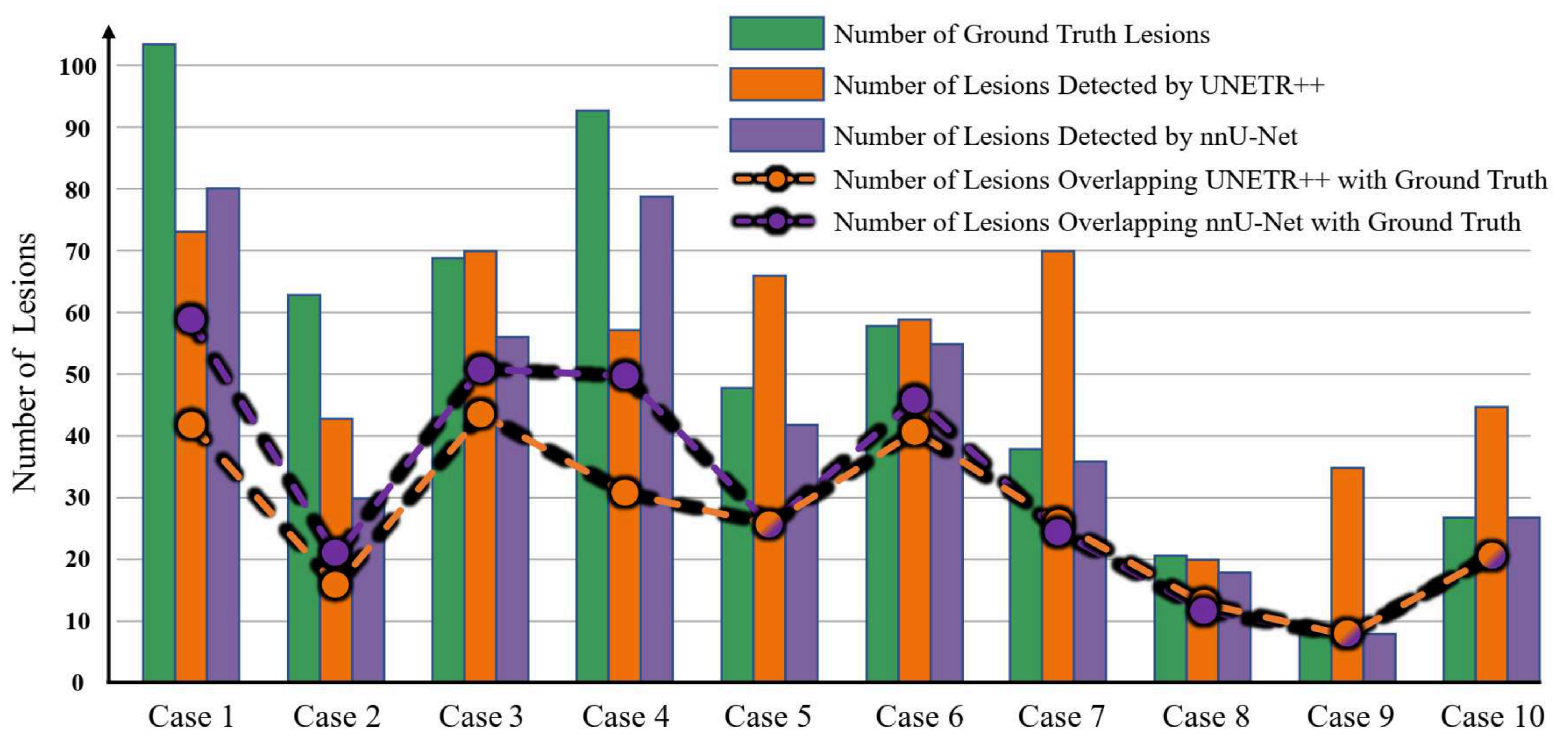
The number of lesions in each test case, the lesions detected by UNETR++ and nnU-Net, and the predicted lesions that overlap with the ground truth are displayed.

The values reported in Figure 6 and Figure 7 are based on the number of pixels. Given that the number of lesions varies across cases, Figure 8 shows the number of lesions per network and the number of overlapping lesions between the predicted and ground truth in each test case.

For further lesion-wise assessments, Table 3 presents detailed performance metrics for segmentation models, including Lesion-wise F1/Dice, lesion-wise detection rate (LDR), and two other surface-based metrics, HD95 and ASD, on True predicted lesions and their corresponding ground truth lesions. The number of detected false-positive lesions has been calculated, and it is shown that UNETR++ identifies an average of 25.9 false-positive lesions per case, whereas nnU-Net identifies 8.1. This table also reports the bootstrap results, together with the corresponding 95% CIs, computed using 1000 iterations resampled with replacement at the patient level within the independent test cohort (*N* = 10). These CIs are reported alongside the mean and standard deviation to comprehensively present the overall segmentation and classification performance. A smaller difference between these bounds reflects a narrower interval, implying greater stability of the estimate and reduced uncertainty.

**Figure 9:**
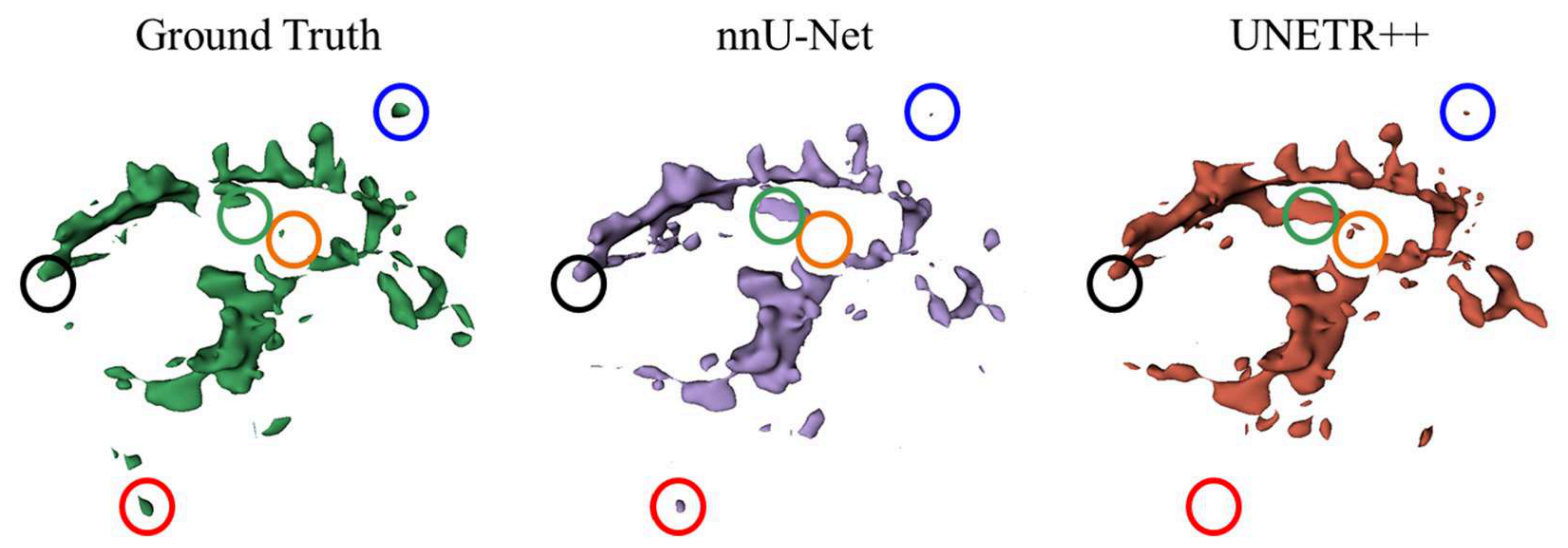
Visual comparison of the ground truth and the segmentation results of both algorithms. Highlighted regions mark differences between the models and the ground truth.

For qualitative assessment, Figure 9 illustrates an example data case, along with the segmentation outputs from both networks. The annotated circles highlight the differences between the two algorithms and their discrepancies relative to the ground truth.

**Figure 10:**
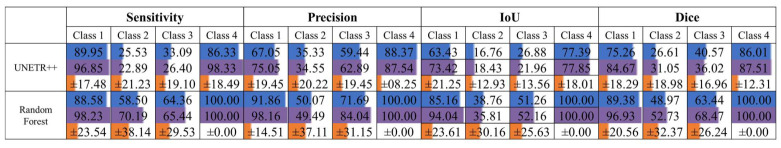
Results for sensitivity, precision, IoU, and Dice for the two algorithms when given binary ground truth as input. The mean (blue), median (purple), and std (orange) are shown separately for each class.

In the next step, MS lesions were classified into four classes using both ML and DL algorithms. On the other hand, seven ML algorithms, including Random Forest, which achieved the best overall performance, were implemented for comparison against the UNETR++ as the DL classifier. To maximize the predictive capabilities of this top-performing Random Forest model, its hyperparameters were rigorously tuned over 1000 trials using the Optuna framework under an 8-fold cross-validation scheme. The resulting optimal configuration utilized an ensemble of 288 independent decision trees guided by Shannon entropy, with structural constraints including a maximum depth of 20 to ensure robust generalization and validation stability. Figure 10 reports the sensitivity, precision, IoU, and Dice metrics for the two classification algorithms when a binary ground truth is used as input.

Given that, in the best condition, the classification network receives binary ground truth as input. When the classification network’s input is the segmentation network’s output, we normalize the segmentation network’s output to align with the ground truth defined in optimal conditions. The results of this normalization are summarized in Table 4. Table 5 also represents an analogous set of statistical computations to those outlined in Table 4, modified here to incorporate patient-level bootstrap resampling.

**Figure 11:**
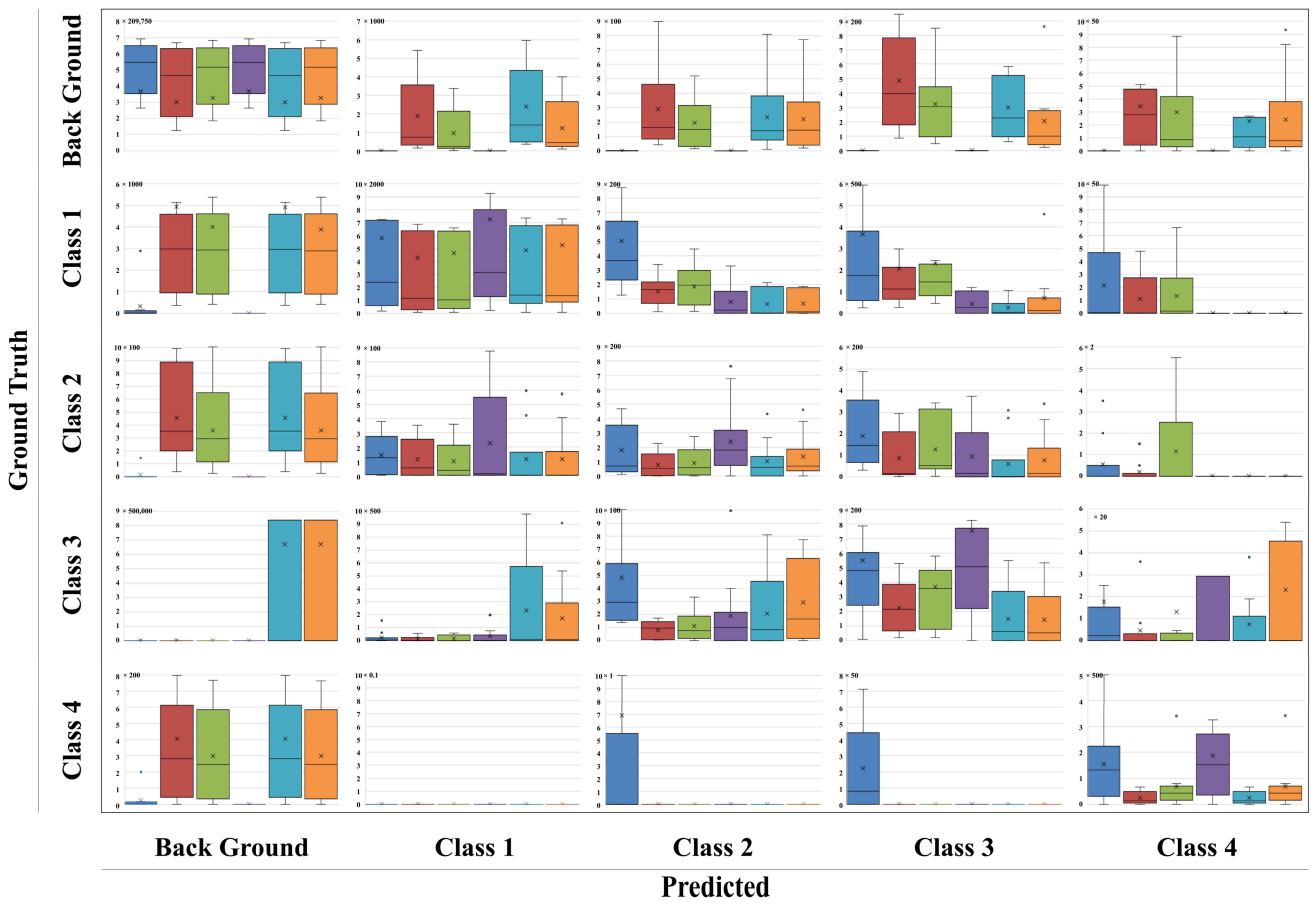
Confusion matrix for classification networks across six conditions. UNETR++ network (ground truth input (blue), UNETR++ input (red), and nnU-Net input (green)), Random Forest network (ground truth input (purple), UNETR++ input (cyan), and nnU-Net input (orange)).

Figure 11 displays the confusion matrices for each of the six situations, demonstrating which method has the most errors in assigning each class.

**Figure 12:**
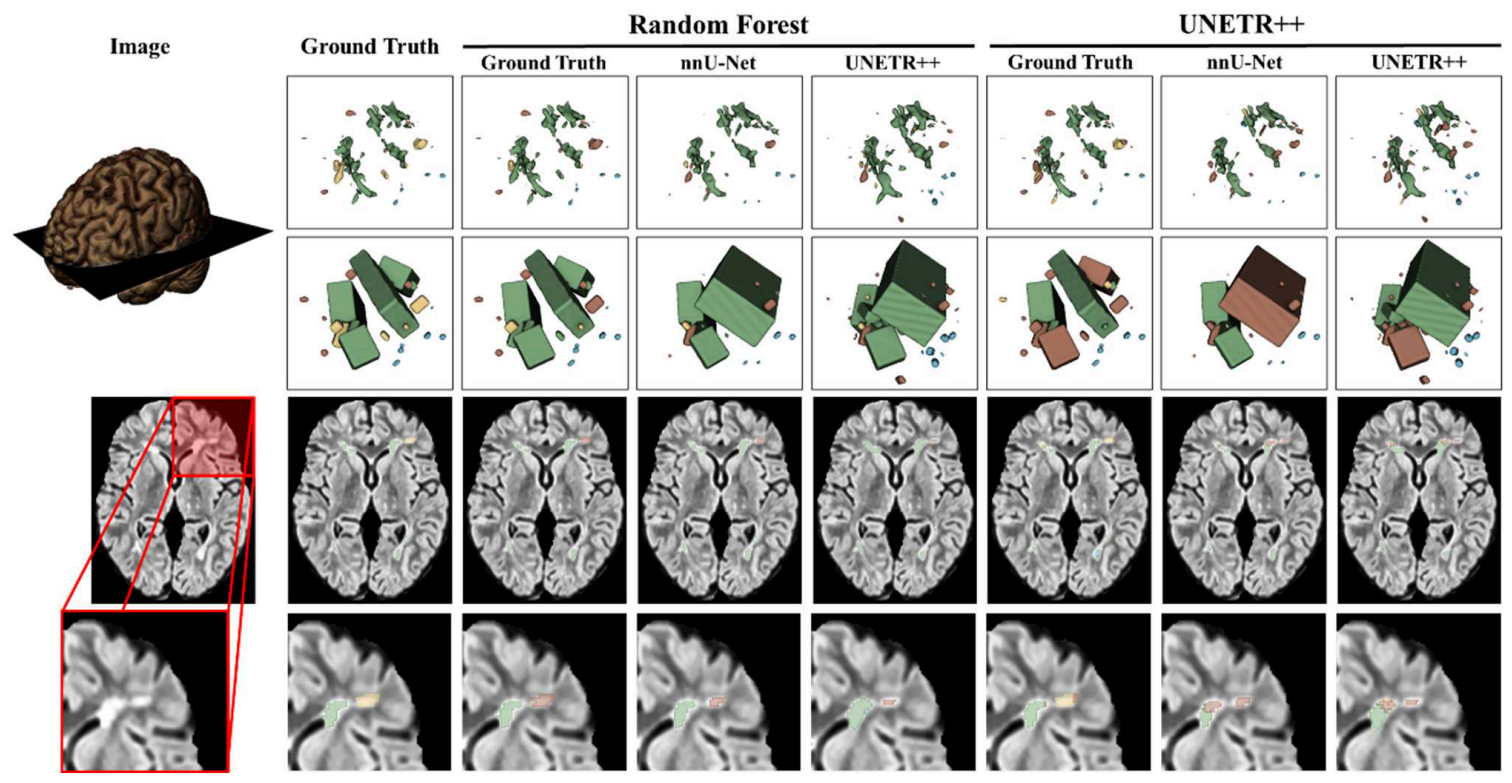
The first row shows the classification results for different conditions in case 7, and the second row shows the optimal box in those results. The third row shows the classification results for a slice of the image, and the last row shows the magnified region.

Finally, since the location of the lesions may be more important than their exact boundaries, the lesions were boxed, and the results for one case are shown in Figure 12.

## 5. Discussion

Automatic MS lesion segmentation and classification constitute critical steps toward faster, more accurate diagnosis, treatment planning, and prognosis assessment. Precise delineation of lesion boundaries is essential in clinical practice. Similarly, determining the lesions’ location, whether by pinpointing their exact location or identifying the anatomical brain region they occupy, is equally important. Although several U-Net-based networks and ML approaches have been proposed for segmentation and classification, hybrid methodologies have demonstrated promising results in related applications such as brain tumor analysis. Therefore, in this study, we proposed a hybrid pipeline for joint segmentation and classification [51] and evaluated multiple architectural combinations. For segmentation, two U-Net-based networks, nnU-Net and UNETR++, were employed. Both models were trained on a multiclass labeled dataset. The diversity of classes was ensured before testing the models by selecting samples containing all four lesion classes, with varying lesion counts (Table 2). Notably, analysis of cases 2 and 4 revealed that a higher pixel count does not necessarily correspond to a greater number of lesions, suggesting variability in lesion size. While our test set is limited to 10 patient cases, the evaluation leverages highly granular voxel-level and lesion-level resolutions to provide a detailed, localized assessment of model performance. Our patch-based 3D framework processes a large volume of distinct spatial regions, providing a comprehensive representation of structural features. Furthermore, the test dataset exhibits substantial clinical heterogeneity, encompassing hundreds of distinct lesion components across the four anatomical zones. This structural variety provides a wide range of performance estimates across diverse lesion sizes and spatial distributions, mitigating the risk of performance metrics being skewed by a single patient phenotype. Quantitative results (Figure 6) demonstrate that nnU-Net provides higher mean values across all evaluation metrics, with improvement ranging from 4.8% to 12.8%. This performance gain may be attributed to its residual connections. Although nnU-Net exhibits slightly higher variance in Dice and IoU (0.6% and 2.1%, respectively) than UNETR++, this difference is forgivable. Case-wise analysis, as shown in Figure 7, further confirms the superiority of nnU-Net across all metrics. Dice and IoU criteria follow consistent trends; however, deviations are observed in precision and sensitivity for cases 5 and 9, where UNETR++ outperforms nnU-Net. Detailed inspection indicates that UNETR++ tends to identify additional regions as lesions that are not annotated in the ground truth, as seen in cases 3, 6, 7, and 9. Some of these regions appear hyperintense and may correspond to true MS lesions, which could cause the observed variance.

Further comparison based on lesion count (Figure 8) shows that although UNETR++ predicted more lesions overall, nnU-Net achieved a higher number of correct predictions (dashed line) in most cases, including 5, 9, and 10. This indicates that UNETR++ yields more false positives, likely because its attention mechanism overemphasizes certain regions. Consequently, UNETR++ shows lower precision. Additionally, both models predicted the same number of lesions in cases 5 and 9; however, pixel-wise analysis reveals that UNETR++ tends to generate larger lesion regions. Furthermore, qualitative assessment (Figure 9) highlights complementary strengths of the two models. However, as shown in the blue circle, nnU-Net produces smaller lesions than the ground truth. Finally, our results suggest that nnU-Net achieves effective lesion localization and detection, while UNETR++ demonstrates superior performance in capturing lesion boundaries and volumes. Both models also identified lesions not included in the ground truth, which may reflect limitations in the reference annotations. If confirmed, this would further improve the perceived precision of both methods, particularly UNETR++.

The high false-positive rate of UNETR++ is clearly demonstrated in Table 3. On average, UNETR++ produces 25.9 false lesions per subject, which is substantially higher than the false-positive rate observed with nnU-Net. Even when both models achieve comparable lesion-detection rates (e.g., cases 5 and 10), nnU-Net performs better across most evaluation metrics. This improvement is due to its lower false detection rate, which directly affects the Dice coefficient.

However, in case 9, UNETR++ performs poorly in most cases but attains slightly better boundary-based metrics. However, its lesion-wise Dice score drops by approximately 60% compared to nnU-Net, which achieves a perfect 100%. A similar discrepancy appears in case 7, where UNETR++ yields a low Dice score but reports lower HD95 and ASD values. These observations support the interpretation that nnU-Net’s segmentations are highly localized, particularly in challenging cases.

Some of these inconsistencies may also be attributed to dataset outliers, which make certain subjects behave differently. For example, the unusually large HD95 value in case 7 suggests atypical lesion geometry or annotation variability. Despite these exceptions, the overall results in Table 3 indicate that nnU-Net performs better across multiple metrics and subjects. The lesion-wise evaluation further confirms that nnU-Net generally produces boundary predictions that align well with the ground truth.

The bootstrap results indicate that nnU-Net performs better overall than UNETR++, as it consistently shows higher mean values and wider lower and upper CI bounds across almost all metrics. Although UNETR++ exhibits slightly higher values only in the HD mean and ASD mean upper bounds, these differences are likely influenced by outliers. Given that nnU-Net also achieves lower upper-band variance and a smaller IQR, the apparent advantage of UNETR++ on these metrics is negligible. In terms of CIs’ behavior, both networks show relatively narrow intervals, indicating stable performance. However, because nnU-Net almost always maintains higher mean values and higher lower CI bounds, it provides more reliable performance across patients. Therefore, even though each network occasionally outperforms the other in isolated CI comparisons, nnU-Net can be regarded as the more ideal and consistently superior model.

In the next step, for the classification stage, we investigated and compared ML and DL approaches following the binary segmentation. Among the evaluated ML methods, Random Forest achieved the best results due to its suitability for complex classification and was therefore selected for further comparison [52]. For DL, UNETR++ was chosen as the representative classifier. Under ideal conditions (i.e., using ground truth segmentation; Figure 10), Random Forest significantly outperforms UNETR++ across most metrics and classes, with improvements exceeding 12% in several cases. The only exception is the sensitivity of first-class lesions, where UNETR++ demonstrated slightly better performance (1.37%). Random Forest achieves near-perfect classification of fourth-class lesions, likely due to their spatial separation, as captured by the distance-based features. Also, additional assessments indicated that the variance is primarily due to a small number of misclassified lesions, as evidenced by median values that are comparable to or exceed the mean.

In practical scenarios, where segmentation outputs are used instead of ground truth (Table 4), we normalized classification results using the binary segmentation outputs of nnU-Net and UNETR++. Also, we found that Random Forest continues to outperform UNETR++. The best overall performance is achieved when nnU-Net is used for segmentation and Random Forest for classification. However, DL methods typically require larger datasets, and their performance may improve with more training data. Table 5 provides the bootstrap results for the four evaluated metrics. These results confirm the stability of the proposed model, as the bootstrapped values remain close to the original calculations. The table also shows that the combination of nnU-Net and Random Forest performs best overall, with higher mean values and higher upper and lower CI bounds across most metrics. However, both tables indicate that UNETR++ achieves slightly higher sensitivity for first-class lesions by approximately two percent, which may be attributed to the proximity of these lesions to second-class lesions, making them more challenging for distance-based classification methods. To further investigate classification behavior, the confusion matrix in Figure 11 was analyzed. The first three bars in each cell represent the results of UNETR++, and the second three bars demonstrate the random forest’s results.

Under ideal conditions, when ground truth is provided, both approaches perform similarly in distinguishing between background and lesion classes, confirming that classification models can achieve high accuracy when segmentation is flawless. However, segmentation errors significantly degrade classification performance, particularly in third-class lesion prediction using Random Forest. In contrast, UNETR++ demonstrates greater robustness to imperfect segmentation in this class. This observation is supported by the background predictions column, where many expected third-class lesions are misclassified as background. On the positive side, Random Forest avoids misclassifying first- and second-class lesions as fourth-class lesions, and no fourth-class lesions are misclassified as any other class. Moreover, when ground truth is available, Random Forest correctly classifies nearly all lesions, unlike UNETR++. It is worth noting that our algorithms achieved substantially improved results even without considering permutation, compared to the two state-of-the-art methods that won the IAI2023 challenge [33].

A direct, head-to-head comparison is particularly challenging because most existing studies use different datasets and lack the specific, annotated data required for anatomical lesion classification. Nevertheless, to provide the requested benchmarking, we have compared our performance against the top-performing teams from the IAI2023 challenge, which constitutes the most relevant and recent baseline available for this dataset. Table 6 compares the performance of our proposed model with that of the first- and second-place teams in the challenge.

As shown in Table 6, our proposed pipeline significantly outperforms the established baselines across all four classes. Most notably, our model achieved sensitivity scores ranging from 61.27% to 85.03%, compared with the challenge’s best algorithm, which achieved sensitivities between 14.40% and 22.50%. This performance gain is primarily due to the hybrid integration of the nnU-Net segmentation framework and the Random Forest classification logic, which effectively addresses the inter-class heterogeneity that previous methods struggled to handle. However, a more specific comparison would be possible if the prior article had reported variance metrics. A qualitative assessment has been executed for classification (Figure 12) and emphasizes the importance of spatial context. Lesions located in proximity but belonging to different classes remain challenging to distinguish.

Regarding this point, clear and precise labels are necessary; this underscores the need for high-quality annotations, ideally verified by expert neuroradiologists. Future work could explore incorporating spatial priors or post-processing techniques to improve class separability. Furthermore, evaluating the proposed pipeline on larger and more diverse datasets would provide insights into its generalizability.

## 6. Conclusions

In this study, we proposed a hybrid pipeline for joint segmentation and classification of MS lesions to facilitate MS diagnosis and treatment planning. According to the results, nnU-Net outperforms UNETR++ in the segmentation task, with lower false-positive rates and higher true-positive detection. Meanwhile, Random Forest provides superior classification performance compared to DL approaches and other ML algorithms, particularly when combined with nnU-Net segmentation. Improved segmentation results lead to improved classification performance. Overall, the findings highlight the strong dependency of classification performance on segmentation quality. Finally, the combination of nnU-Net for segmentation and Random Forest for classification yields the most reliable results. Future work should focus on incorporating larger datasets and improving annotation quality to enhance classification performance and generalizability further.

## Figures and Tables

**Table 1:** Information on the intensity values for each lesion class, ranging from 0 to 1.

	Class 1 (Periventricular)	Class 2 (White Matter)	Class 3 (Juxtacortical)	Class 4 (Infratentorial)
Minimum	0.3	0.41	0.41	0.38
First Quartile	0.61	0.55	0.59	0.53
Median	0.66	0.61	0.64	0.56
Third Quartile	0.7	0.67	0.69	0.59
Maximum	0.82	0.8	0.805	0.68

**Table 2:** Ten cases were selected for testing the models. The number of pixels and lesions (values in parentheses) for each case across four classes is reported.

	Case 1	Case 2	Case 3	Case 4	Case 5	Case 6	Case 7	Case 8	Case 9	Case 10
**Class 1**	4327 (31)	3911 (29)	3135 (21)	41,030 (23)	3228 (29)	9683 (19)	7844 (9)	459 (6)	1062 (5)	1434 (12)
**Class 2**	908 (24)	205 (14)	1095 (29)	203 (5)	177 (5)	1547 (25)	847 (10)	511 (5)	241 (2)	126 (4)
**Class 3**	1350 (37)	548 (18)	663 (12)	4081 (52)	652 (8)	774 (12)	297 (12)	803 (10)	72 (1)	431 (5)
**Class 4**	834 (11)	129 (2)	557 (7)	1632 (13)	562 (6)	177 (2)	243 (7)	0	0	642 (6)

**Table 3:** Overall segmentation network performance and lesion-wise metrics. For each case, the first (white) row presents the nnU-Net results, whereas the second (gray) row corresponds to the UNETR++ outputs. The bold values indicate the best results for the two networks mentioned. Note: The F1/Dice column represents the lesion-wise F1/Dice score, which is calculated based on the lesion counts (TP, FP, FN) rather than voxel overlap.

No. Case		GT Lesion Count	Pred Lesion Count		TP	FP	FN	LDR (%)	F1/Dice (%)	HD95 (mm)			ASD (mm)		
										Mean	Median	IQR	Mean	Median	IQR
case 1		103	80		**59**	**9**	**44**	**57.28**	**69.01**	**2.39**	**1.60**	**1.60**	**0.87**	**0.44**	**0.71**
			73		42	23	61	40.78	50.00	3.00	2.02	1.84	1.25	0.84	0.97
case 2		63	30		**21**	**3**	**42**	**33.33**	**48.28**	**2.74**	**2.26**	**1.10**	**1.06**	**0.73**	**0.60**
			43		16	24	47	25.40	31.07	2.98	2.53	1.36	1.30	1.00	0.82
case 3		69	56		**51**	**8**	**18**	**73.91**	**79.69**	4.04	**1.60**	**1.53**	1.85	**0.50**	**0.48**
			70		44	32	25	63.77	60.69	**3.98**	2.23	3.21	**1.79**	0.94	1.07
case 4		93	79		**50**	**19**	**43**	**53.76**	**61.73**	**6.36**	**1.79**	**1.50**	**2.74**	**0.73**	**0.74**
			57		31	18	62	33.33	43.66	7.56	2.58	4.29	3.31	1.17	1.56
case 5		48	42		**26**	**12**	**22**	**54.17**	**60.47**	**4.47**	**2.21**	**7.31**	**1.54**	**0.71**	**1.78**
			66		**26**	40	**22**	**54.17**	45.61	5.34	3.17	7.34	2.01	1.05	2.39
case 6		58	55		**46**	**9**	**12**	**79.31**	**81.42**	**5.03**	**1.79**	**1.60**	**1.86**	**0.64**	**0.54**
			59		41	20	17	70.69	68.91	5.07	1.96	2.45	2.14	0.79	0.71
case 7		38	36		25	**13**	13	65.79	**65.79**	16.32	1.79	5.51	6.31	0.79	1.74
			70		**26**	44	**12**	**68.42**	48.15	**13.44**	**1.78**	**2.18**	**5.09**	**0.71**	**0.79**
case 8		21	18		12	**5**	9	57.14	**63.16**	**1.32**	**1.13**	**0.64**	**0.43**	**0.28**	**0.24**
			20		**13**	8	**8**	**61.90**	61.90	3.25	1.96	2.17	1.15	0.77	1.41
case 9		8	8		**8**	0	**0**	**100.00**	**100.00**	1.22	1.13	0.45	**0.46**	**0.41**	0.21
			35		**8**	27	**0**	**100.00**	37.21	**1.10**	**0.97**	**0.43**	0.49	0.51	**0.12**
case 10		27	27		**21**	**3**	**6**	**77.78**	**82.35**	**2.16**	**1.60**	**1.28**	**0.85**	**0.58**	**0.47**
			45		**21**	23	**6**	**77.78**	59.15	2.78	2.40	1.41	1.22	1.06	0.80
Bootstrap (95% CIs)	Mean	53 35 72	Mean	43	**32**	**8**	**21**	**65.25**	**71.19**	**4.60**	**1.69**	**2.25**	**1.80**	**0.58**	**0.75**
			Lower	29	**22**	**5**	**12**	**55.37**	**63.55**	**2.48**	**1.49**	**1.11**	**0.95**	**0.48**	**0.47**
	Lower		Upper	58	**43**	**12**	**31**	**75.91**	**79.76**	7.37	**1.91**	**3.66**	2.88	**0.68**	**1.10**
			Mean	54	27	26	26	59.62	50.63	4.85	2.16	2.67	1.97	0.88	1.06
	Upper		Lower	44	20	20	13	47.00	43.95	3.09	1.81	1.65	1.29	0.77	0.72
			Upper	64	34	32	41	72.24	57.52	**7.02**	2.46	3.88	**2.79**	0.99	1.42

**Table 4:** Results for mean sensitivity, precision, IoU, and Dice for two classification algorithms when the classification network is fed the outputs of the binary segmentation networks.

Normalized Based on the Best GT Result					Class 1	Class 2	Class 3	Class 4
Dice	Segmentation	UNETR++	Classification	UNETR++	54.06	28.25	30.90	28.92
				Random Forest	63.13	45.46	45.24	31.94
		nnU-Net		UNETR++	57.41	38.38	45.22	53.54
				Random Forest	**68.99**	**54.01**	**61.97**	**56.55**
IoU		UNETR++		UNETR++	40.67	20.32	21.99	21.55
				Random Forest	48.95	35.66	34.18	24.19
		nnU-Net		UNETR++	45.06	28.41	34.30	41.72
				Random Forest	**56.65**	**41.78**	**51.14**	**44.43**
Sensitivity		UNETR++		UNETR++	74.00	30.98	26.78	46.48
				Random Forest	71.95	54.30	53.82	62.90
		nnU-Net		UNETR++	**86.83**	37.75	37.88	72.47
				Random Forest	85.03	**61.27**	**72.38**	**80.40**
Precision		UNETR++		UNETR++	48.52	27.48	40.98	37.36
				Random Forest	60.38	36.70	46.65	37.38
		nnU-Net		UNETR++	47.76	35.77	54.66	51.02
				Random Forest	**61.12**	**55.00**	**54.85**	**51.05**

Note: Bold values indicate the best results achieved by each algorithm under specific conditions.

**Table 5:** Report the patient-level bootstrapped means for sensitivity, precision, IoU, and Dice for the two classification algorithms. In each cell, the first value represents the bootstrapped mean, while the values in parentheses indicate the lower and upper bounds of the 95% confidence interval.

Normalized Based on the Best GT Result					Class 1	Class 2	Class 3	Class 4
Dice	Segmentation	UNETR++	Classification	UNETR++	53.93 (46.67–62.24)	28.57 (22.58–32.35)	31.75 (28.26–35.06)	29.00 (13.00–**100**)
				Random Forest	62.92 (60.00–68.37)	44.90 (35.48–50.00)	46.03 (43.48–49.35)	32.00 (15.00–**100**)
		nnU-Net		UNETR++	57.30 (48.00–67.35)	38.78 (32.26–39.71)	46.03 (41.30–49.35)	54.00 (39.00–**100**)
				Random Forest	**69.66 (62.67–75.51)**	**53.06 (54.84–52.94)**	**61.90 (60.87–66.23)**	**57.00 (42.00–100)**
IoU		UNETR++		UNETR++	41.18 (33.33–47.92)	20.51 (18.18–22.81)	21.57 (19.44–24.24)	22.00 (08.00–**100**)
				Random Forest	49.41 (44.93–54.17)	35.90 (31.82–38.60)	35.29 (33.33–36.36)	24.00 (09.00–**100**)
		nnU-Net		UNETR++	44.71 (36.23–54.17)	28.21 (27.27–28.07)	35.29 (30.56–36.36)	42.00 (25.00–**100**)
				Random Forest	**56.47 (50.72–63.54)**	**41.03 (45.45–40.35)**	**50.98 (47.22–54.55)**	**44.00 (27.00–100)**
Sensitivity		UNETR++		UNETR++	74.16 (71.23–77.78)	31.03 (25.71–35.37)	22.97 (16.39–28.74)	46.00 (28.00–**100**)
				Random Forest	71.91 (68.49–76.77)	55.17 (45.71–**58.54**)	47.30 (42.62–51.72)	63.00 (42.00–**100**)
		nnU-Net		UNETR++	**86.52 (84.93–88.89)**	37.93 (31.43–41.46)	32.43 (24.59–40.23)	72.00 (58.00–**100**)
				Random Forest	84.27 (80.82–87.88)	**62.07 (68.57–58.54)**	**63.51 (54.10–68.97)**	**80.00 (69.00–100)**
Precision		UNETR++		UNETR++	48.91 (36.59–60.61)	28.00 (20.69–30.56)	40.28 (36.54–46.07)	37.00 (16.00–**100**)
				Random Forest	59.78 (**50.00**–69.70)	36.00 (31.03–41.67)	45.83 (40.38–52.81)	37.00 (16.00–**100**)
		nnU-Net		UNETR++	47.83 (35.37–59.60)	36.00 (34.48–37.50)	54.17 (53.85–57.30)	**51.00 (33.00–100)**
				Random Forest	**60.87 (50.00–71.72)**	**56.00 (55.17–56.94)**	**54.17 (51.92–58.43)**	**51.00 (33.00–100)**

Note: Bold values indicate the best results achieved by each algorithm under specific conditions.

**Table 6:** Comparative Performance Evaluation across Four Anatomical Lesion Classes.

	1st Team			2nd Team			Our Model		
	Evaluation Metrics (%)								
	Dice	Sensitivity	Precision	Dice	Sensitivity	Precision	Dice	Sensitivity	Precision
Class 1	38.4	14.4	51	13.3	13.3	46.8	**68.99**	**85.03**	**61.12**
Class 2	38.4	14.4	18.6	14.6	15.1	13.3	**54.01**	**61.27**	**55.00**
Class 3	36.5	19.9	24.1	27.6	8.2	10.7	**61.97**	**72.38**	**54.85**
Class 4	32.8	22.5	20.7	0.000	0.000	0.000	**56.55**	**80.40**	**51.05**

Note: Bold values indicate the best results achieved by each algorithm under specific conditions.

## Data Availability

The IAI2023 FLAIR MRI dataset and corresponding MS lesion masks are available from the corresponding author upon reasonable academic request. Full dataset details and clinical annotations are described in Reference [33].

## Abbreviations

The following abbreviations are used in this manuscript:

MS	Multiple Sclerosis
DL	Deep Learning
ML	Machine Learning
MRI	Magnetic Resonance Imaging
FLAIR	Fluid-attenuated Inversion Recovery
AI	Artificial Intelligence
CNN	Convolutional Neural Network
DTs	Decision Trees
RFs	Random Forests
SVMs	Support Vector Machines
LSTM	Long Short-Term Memory
CLAHE	Contrast Limited Adaptive Histogram Equalization
FLOPs	Floating-point Operations Per Second
EPA	Efficient Paired Attention
PCA	Principal Component Analysis
CE	Cross Entropy
IoU	Intersection over Union
STD	Standard Deviation
HD95	95th-percentile Hausdorff Distance
ASD	Average Surface Distance
LDR	Lesion-wise Detection Rate
DIS	Demonstrating Dissemination in Space
CIs	Confidence Intervals
IQR	Interquartile Range
